# Supplementary material for: Prevalence of hypertension and associated factors among nurses working in private and public tertiary hospitals in Dar Es Salaam, Tanzania
Source: PLOS Glob Public Health. 2026 Jan 15;6(1):e0005152. doi: 10.1371/journal.pgph.0005152 (PMC12806849; doi:10.1371/journal.pgph.0005152)
Supplement: Attachment — Submitted filename: RESPONSE TO REVIEWERS MATRIX. 17 Nov 2025.pdf [file pgph.0005152.s002.pdf]

TITLE: PREVALENCE OF HYPERTENSION AND ASSOCIATED FACTORS AMONG NURSES WORKING IN PRIVATE AND PUBLIC TERTIARY HOSPITALS IN DAR ES SALAAM, TANZANIA

EDITOR'S COMMENTS AND RESPONSES

COMMENTS	RESPONSE
Please ensure that your Ethics Statement is available in its entirety at the beginning of your Methods section, under a subheading 'Ethics Statement'.	We thank the reviewer for this comment. We have added the complete Ethics Statement at the beginning of the Methods section under a subheading titled "Ethics Statement", in accordance with journal guidelines.<br>Page 4.
We note that your Data Availability Statement is currently as follows: "All relevant data are fully contained within the manuscript. No additional datasets were generated or analyzed during the current study. Please confirm at this time whether or not your submission contains all raw data required to replicate the results of your study. Authors must share the "minimal data set" for their submission	We acknowledge the editor's comment regarding the Data Availability Statement. During resubmission, we will revise the statement to clarify data accessibility and ensure compliance with PLOS data-sharing requirements. The anonymized minimal dataset used to generate the study findings has been uploaded to a public data repository, and the corresponding link will be provided in the revised manuscript.

REVIEWER 1 COMMENTS AND RESPONSES

SECTIONS	COMMENT	RESPONSES
**ABSTRACT**	The title and abstract in a clear sentences, but the abstract must not be more than 300 words, precisely describe the main aspect of this study.	Thank you for the comment, we have reduced the abstract to fit the recommendation
**INTRODUCTION**	The introduction is well composed, updated and informative, mentioning recent publications about the problem of investigation.	Thank you
**METHODS**	Methods are clearly described	Thank you
**RESULTS**	Results are important and relevant	Thank you
	The figures and tables are clear and legible	Thank you
**DISCUSSION**	Discussion is clear, well-structured and designed, correlates with the results	Thank you
**CONCLUSION**	Conclusions correlate to the results	Thank you

REVIEWER 2 COMMENTS AND RESPONSES

SECTION	COMMENTS	RESPONSES
**INTRODUCTION**	I find this to be a well-structured and relevant cross-sectional study that addresses an important occupational health issue in a low-resource setting	Thank you
	The manuscript is clearly written	Thank you
**METHODS**	Methods are appropriately described, and the sampling strategy is justified given the context	Thank you
	The use of multivariable regression to identify associated factors is a strength, and several findings—such as the strong associations with age, BMI, physical inactivity, and work-related stress—are consistent with existing literature	Thank you
**RESULTS**	The prevalence of hypertension (28.9%) among nurses is concerning and highlights a significant public health problem	Thank you
	Additionally, some confidence intervals in the results are unusually wide (e.g., for physical inactivity and poor diet), which may reflect small subgroup sizes or model instability and should be interpreted cautiously.	We acknowledge that some confidence intervals were wide. This likely reflects the small number of hypertensive cases within certain exposure subgroups and the relatively low variability within some exposure categories. The multivariable logistic regression model was checked for multicollinearity and overall fit, and no evidence of model instability was found. This limitation has been noted in the Discussion.<br>Page 17-18.
**LIMITATION**	Focus on only two urban tertiary hospitals, which affects generalizability	This limitation has been acknowledged and clearly stated in the revised manuscript. We have also suggested that future studies include a wider range of healthcare settings and geographical areas to enhance generalizability.<br>Page 17-18.
	Reliance on self-reported data for behavioral factors introduces potential bias	We thank the reviewer for highlighting this point. The information was collected using a structured and validated questionnaire, such as STEPS survey tool, GPAQ and Stress tool in which responses were scored to classify participant's behaviors. This approach helped standardize the data and reduce subjectivity. We also recognize the potential for recall and social desirability bias, as noted in our limitations section, and we have clarified this in the revised manuscript to ensure consistency.<br>Page 17-18.

**CONCLUSION**	Despite these limitations, the study provides valuable insights and practical recommendations for workplace interventions aimed at reducing hypertension risk among nurses in Tanzania.	Thank you

REVIEWER 3 COMMENTS AND RESPONSES

SECTIONS	COMMENT	RESPONSES
**INTRODUCTION**	This manuscript explores the prevalence of hypertension among nurses in public and private tertiary hospitals in Dar es Salaam, Tanzania, and examines sociodemographic, behavioral, and occupational risk factors. The topic is important, timely, and relevant for occupational and public health in sub-Saharan Africa. The use of standardized blood pressure measurement methods is a strength, and the inclusion of both private and public hospital settings adds useful context. However, several aspects of the study require clarification or revision before the manuscript can be considered for publication.	Thank you
**METHODS AND ANALYSIS**	The cross-sectional design is appropriate for estimating prevalence but does not allow for causal inference. This limitation should be stated more clearly in the discussion.	We thank the reviewer for this valuable observation. We have added relevant text to discuss this limitation.<br>Page 17-18.
	Categorization of continuous variables such as sleep duration, dietary practices, BMI, and stress requires further justification	We thank the reviewer for this observation. The categorization of these variables followed established guidelines. Dietary practices were classified as good or poor based on WHO STEPwise criteria for fruit, vegetable, and salt intake. BMI categories followed WHO standard cut-offs, while work-related stress levels were derived from the American Institute of Stress Workplace Stress Scale. These categorizations are consistent with previous studies and facilitate comparison across research.<br>Page 6.

	The rationale for cutoffs (e.g., ≤5 hours for sleep) should be explained and ideally supported by prior literature.	The rationale for the selected cutoffs (e.g., ≤5 hours for sleep) has now been clarified and supported with relevant literature in the Methodology section. A reference has been added to justify the cutoff used based on previous studies examining sleep duration and hypertension risk.<br>Page 6
**RESULTS**	Logistic regression is correctly applied, but several adjusted odds ratios show very wide confidence intervals (e.g., AOR 3.23; 95% CI 2.39–15.60), suggesting sparse data in subgroups or possible overfitting	We acknowledge that some confidence intervals were wide. This likely reflects the small number of hypertensive cases within certain exposure subgroups and the relatively low variability within some exposure categories. The multivariable logistic regression model was checked for multicollinearity and overall fit, and no evidence of model instability was found. This limitation has been noted in the Discussion.<br>Page 17-18.
	Please provide information on the number of events per variable included in the models	This information is available in the second column of Tables 2, 3 and 4.<br>Pages 10-13
**DISCUSSION**	The interpretation of education level as a risk factor is inconsistent: results tables indicate higher odds among nurses with degree-level education, while the discussion suggests the opposite. This discrepancy should be resolved.	We appreciate the reviewer's observation. The inconsistency between the results table and the discussion was due to a typographical error in the table. The table has been corrected to accurately reflect the findings, which show that nurses with lower levels of education had higher odds of hypertension. This correction resolves the discrepancy and aligns the results with the discussion.<br>Page 10.
**LIMITATIONS**	The limitations section should be expanded. Restriction of the study to only two tertiary hospitals in an urban area, limiting external validity and generalizability.	We appreciate the reviewer's valuable comment. We have expanded the Limitations section to acknowledge that the study was restricted to two tertiary hospitals in an urban area, which limits the external validity and generalizability of the findings. We have also noted that future studies should include a broader range of healthcare facilities and geographical settings to enhance the representativeness and applicability of the results.<br>Page 17-18.

	Potential residual confounding from unmeasured variables (e.g., shift type, socioeconomic status, other comorbidities)	We acknowledge that, as with all observational studies, residual confounding cannot be fully excluded, although we adjusted for major known confounders such as sex and Age. Unmeasured or imperfectly measured factors, such as shift changes and socioeconomic status, may influence this study.
	Heavy reliance on self-reported measures (diet, stress, alcohol consumption, sleep), which introduces recall and social desirability bias	We thank the reviewer for highlighting this point. The information was collected using a structured and validated questionnaire, such as STEPS survey tool, GPAQ, and Stress tool in which responses were scored to classify participants' behaviors. This approach helped standardize the data and reduce subjectivity. We also recognize the potential for recall and social desirability bias, as noted in our limitations section, and we have clarified this in the revised manuscript to ensure consistency. Page 17-18.
**DATA AVAILABILITY**	An anonymized dataset containing individual-level data (demographics, blood pressure, behavioral and occupational variables) should be deposited in a public repository such as Dryad, OSF, or Figshare, or provided as supplementary files. This step is necessary for reproducibility.	We appreciate the reviewer's comment regarding the provision of individual-level data for reproducibility. We have prepared an anonymized dataset containing participant demographics, blood pressure measurements, and behavioral and occupational variables. This dataset will be deposited in Figshare.
**WRITING AND PRESENTATION**	The manuscript is overall intelligible and written in standard English, but there are minor grammatical errors, occasional awkward phrasing, and some unnecessarily long sentences. A careful language revision would improve clarity	We appreciate the reviewer's input and have carefully edited the manuscript to improve sentence structure, enhance flow, and correct language issues, ensuring the text is more readable and clearly conveys the study findings.
	Variables are informative but could be streamlined to enhance readability. For example, grouping categories or collapsing less relevant variables could make the results more concise and accessible.	Thank you for the suggestion. We have reviewed the variable structure and streamlined key categories to enhance clarity and readability. Specifically, depression status has been consolidated into Moderate to Severe, Mild, and None. Similarly, the variable for the number of patients attended per day has been grouped into three meaningful categories: *>15*, *6–15*, and *1–5*. These revisions ensure that variables remain informative while presenting results in a more concise and accessible format. Page 13.

REVIEWER 4 COMMENTS AND RESPONSES

SECTIONS	COMMENT	RESPONSES
**INTRODUCTION**	The manuscript is generally very insightful and addresses a very pertinent and interesting topic	Thank you
**METHODOLOGY**	The authors should kindly explain what they meant by moderate activity or vigorous activity, and whether it was self-reported by the study participants or observed the researchers. This will provide comprehensive understanding, interpretability, and reliability of the findings.	We have clarified the definition of physical activity in the Methods section. Moderate activity was defined as exercises that cause a slight but noticeable increase in breathing or heart rate (e.g., brisk walking, household chores, gardening), while vigorous activity was defined as exercises that lead to a substantial increase in heart rate and breathing (e.g., running, aerobics, heavy lifting). Physical activity data was collected using a modified WHO STEPwise tool.<br>Page 6.
	Kindly specify the type of multivariable regression model used in this study rather the generalized statement ("Multivariable regression models adjusted for age, sex and high cholesterol, were then used to assess these associations".)	We thank the reviewer for this comment. In the revised manuscript, we have specified that multivariable logistic regression analysis was used. This clarification has been updated in the Methods section.<br>Page 7.
	Kindly state the sample size used for this study under the 'methods and materials' section, and how the sample size was determined or calculated	We have revised the Methods and Materials section to clearly describe the sample size calculation. The sample size of 360 nurses was determined using Cochran’s formula for estimating proportions in cross-sectional studies. An estimated prevalence of 40% for hypertension among nurses was used, with a 95% confidence level and 5% margin of error.<br>Page 4.
	The authors should consider reclassifying the BMI from behavioral factors as BMI is an indictor or measure which could be influenced by not only behavior but genetic, physiology or metabolism, and even environment. Your research did not specifically determine the predictors of the various BMI values among the nurses.	We thank the reviewer for this insightful comment. We have reclassified BMI from behavioral factors to the section on sociodemographic and clinical factors, as BMI reflects a clinical determinant.<br>Page 10.
	The authors should report distinctively the number and proportion of HPT that were determine through 'self-reporting of HPT history', the current usage of HPT medication, and those from the use of the sphygmomanometer	We have now included the breakdown of hypertension identification sources in the demographic results section. Specifically, we present the number and proportion of participants identified with hypertension through self-reported history, current antihypertensive

		medication use, and screening using a sphygmomanometer. Results section, page 8.
